# Comparison of COVID-19 Health Risks With Other Viral Occupational Hazards

**DOI:** 10.1177/0020731420946590

**Published:** 2020-08-09

**Authors:** Jean-Pierre Unger

**Affiliations:** 1 Institute of Population Health Sciences, University of Newcastle, Newcastle upon Tyne, United Kingdom

**Keywords:** COVID-19, occupational health, burden of disease, epidemiology

## Abstract

The European Commission periodically classifies viruses on their occupational hazards to define the level of protection that workers are entitled to claim. Viruses belonging to Groups 3 and 4 can cause severe human disease and hazard to workers, as well as a spreading risk to the community. However, there is no effective prophylaxis or treatment available for Group 4 viruses. European trade unions and the Commission are negotiating the classification of the COVID-19 virus along these 2 categories. This article weighs the reasons to classify it in Group 3 or 4 while comparing its risks to those of the most significant viruses classified in these 2 categories. COVID-19 characteristics justify its classification in Group 4. Contaminated workers in contact with the public play an important role in disseminating the virus. In hospitals and nursing homes, they increase the overall case fatality rate. By strongly protecting these workers and professionals, the European Union would not only improve health in work environments, but also activate a mechanism key to reducing the COVID-19 burden in the general population. Admittedly, the availability of a new vaccine or treatment would change this conclusion, which was reached in the middle of the first pandemic.

The European Commission periodically classifies viruses on their occupational hazards – that is, the risks they entail for workers. This classification defines the level of health and safety protection from exposure to viruses at work that workers are entitled to claim. Viruses belonging to Group 3 can cause severe human disease and hazard to workers, as well as a risk of spreading to the community, but there is usually effective prophylaxis or treatment available. Group 4 viruses are similarly defined, except there is usually no effective prophylaxis or treatment available.^1^ The last classification dates October 31, 2019.^2^ The European trade unions and the European Commission are negotiating the classification of the COVID-19 virus along these 2 categories. This article weighs the reasons to classify it in Group 3 or 4 while comparing COVID-19 risks to those of the most significant viruses classified in these 2 categories. Beyond the degree of protection that it justifies, the usefulness of the comparison stems from the fact that it makes it possible to weight various epidemiological and medical indicators from a public health perspective.

## Methodology

We compared the mortality, morbidity, and transmission in the general population and in workers/professionals exposed to the COVID-19 virus to those of viruses belonging to Groups 3 and 4 and having sufficient public health importance to deserve a chapter in 2 well-known handbooks of infectious diseases^3^ and tropical medicine.^4^ We also examined the hypothesis that contaminated workers in contact with the public not only increase COVID-19 transmission and case fatality rate (CFR) in their work environment but also, importantly, in the community.

Effective treatment reduces suffering, sequellae, and CFR. Prophylaxis reduces incidence and particularly R0^5^ in case of human-to-human transmission, and sometimes CFR as well.

Transmissibility is measured by the household secondary attack rate (SAR). The SAR is the probability that an exposed susceptible person develops the disease over the duration of infectiousness in a case of patient. The R0 basic reproduction number of an infection is the expected number of cases directly generated by 1 case in a population where all individuals are susceptible to infection.^6^ R0 is the product of virus transmissibility by the number of contacts over contagiousness period by the duration of this period. The proportion of population to be vaccinated to achieve herd immunity is given by the following formula:

$$P_{T}\;\mathrm{to}\;\mathrm{be}\;\mathrm{vaccinated}\;=\;(1\;-\;1/R0)\;\times \;100$$

R0 is the index at the start of the epidemic at time t0 when collective immunity is zero. Then we talk about R_e_ at time t. For example, for COVID, R0 was between 2 and 3 and is now between 0.5 and 0.7 in Belgium following confinement.^7^

The (population-based) disease-specific mortality is the product of the CFR by the incidence.

We aimed at assessing the public health importance of the considered diseases in Europe, particularly their direct importance for workers and professionals, and the indirect importance of their infection for community health while testing the hypothesis that workers in contact with the public increase the COVID-19 R0 and its CFR in specific milieux and/or the community.

The most important criterion is the existence of effective prophylaxis and treatment. The second is virus presence in Europe. The third is its population-based mortality, followed by the remaining criteria. Because all viruses can contaminate lab workers, their case is not discussed here.

## Results of Epidemiological Comparison of COVID-19 With Other Viruses of European Commission Groups 3 and 4 of Significant Public Health Importance

For each disease, we examine the prevention, R0, incidence, CFR, general mortality, occupational concentration, and importance in Europe.

### COVID-19

**Figure 1.**
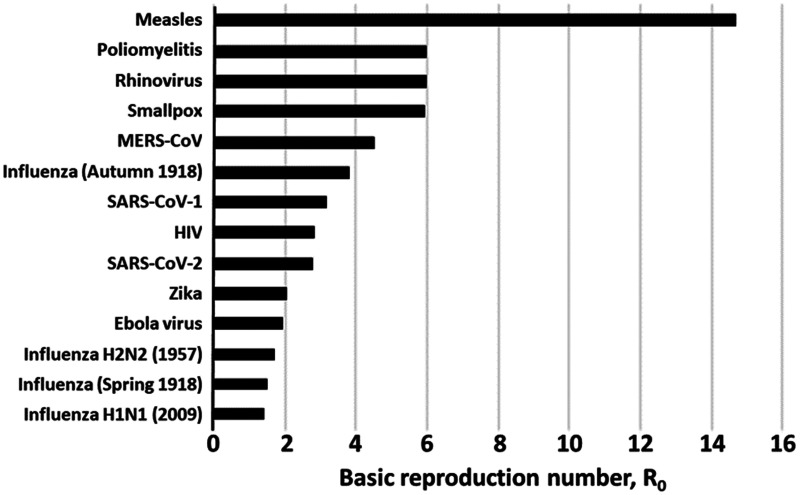
Basic reproduction numbers of selected diseases. Source: Data obtained from Centre for Evidence-Based Medicine.^7^

**Figure 2.**
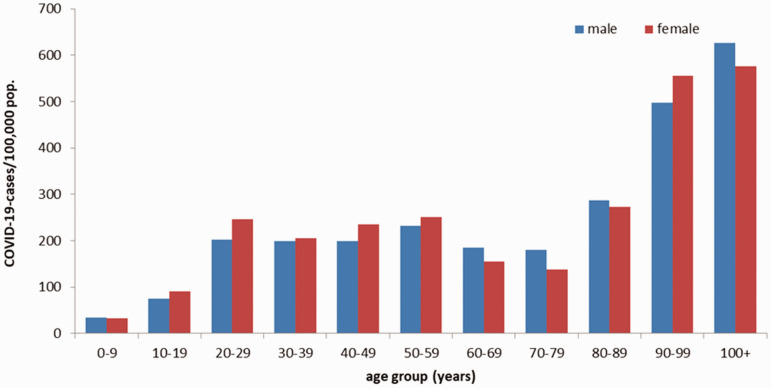
Electronically reported COVID-19 cases/100,000 population in Germany by age group and sex (n = 154,565) for cases with information available as of April 27, 2020, 12:00 a.m., from the “Coronavirus Disease 2019 (COVID-19) Daily Situation Report” of the Robert Koch Institute. Source: Coronavirus Disease 2019 (COVID-19).^23^

- No vaccine is available. Prevention relies on the use of masks,^8^ testing, tracing contacts, and isolation. With regard to treatment, Remdesivir would reduce the sickness episode duration, but its effect on the risk of dying was not demonstrated in 1 study^9^ and, according to another, this effect would be moderate.^10^ Hydroxychloroquine in hospitalized patients with COVID-19 would not be effective,^11^ but it is too early to hold definitive conclusions as it is a combined treatment (hydroxychloroquine + azythromycin) that would have yielded the results in Marseilles and, furthermore, it has been advocated for early uses.
- It is too early to determine the maximum R0 of the epidemic (Figure 1).
- As of May 10, 2020, there were 3,986,119 cases and 278,814 deaths; and 15,685,177 cases and 637,231 deaths as of July 24, 2020.^12^
- The CFR was quite variable according to country (Table 1).^13^ Notice that during an outbreak of a pandemic, the CFR is a poor measure of the mortality risk of the disease because the denominator tends to increase more rapidly as more people are tested. Inter-country comparisons are also misleading, especially between small and large countries, because in large countries the epidemic concentrates regionally^14^ and because of large differences in testing capabilities, leading to the impossibility of knowing the actual numbers of cases, most of them being asymptomatic. Excess deaths (as a percent above normal, established on the average of 5 previous years) is a better indicator.
- Early testing, tracking, contact tracing, isolation, and quarantine explain part of the impact in Germany (3% excess deaths against 72% and 56% in the United Kingdom and Spain, respectively; see Table 1), as do the number of intensive care unit beds per 100,000 population (33.9 in Germany, 15.9 in Belgium, 12.5 in Italy, and 9.7 in Spain).^15^ Hypothetically, this difference could explain why nursing home residents were more easily hospitalized in Germany: The proportion of COVID-19 deaths in nursing homes would increase when the capacity of intensive care units is reduced.
- As with CFR, it is too early to compute the disease-specific mortality rate. However, it can already be observed that mortality in the countries where it is the highest is explained by poorer planning of epidemic control (tracing, testing, isolation, masks) and by a hospital system and nursing homes whose resources have been cut by austerity policies or that are insufficiently accessible because of the commercial financing of health systems^16^ – good planning possibly compensating partially for the other factors. Geographical, demographical, social, economic, political, and cultural variations from country to country can also explain part of the differences in the epidemic transmission (R0), thereby affecting incidence and mortality.
- What are the reasons to believe that workplaces are important to the dissemination of COVID-19 in the general population? Health care^17^ and nursing home workers are well-known disseminators in high-risk populations. Sixty-one percent of people with COVID-19 investigated in New Zealand clusters were in work environment clusters (Table 2).^18^ In Belgium, as of May 5, 53% of deaths occurred in residential homes for the elderly, against 47% in hospitals.^19^ Moreover, the CFR in residential homes for the elderly was 54.7%. (In order to be as complete as possible in reporting the pandemic, the Belgian government includes suspected cases in its deaths count. By April 15, the total number of reported deaths since the beginning of the pandemic was 4,440. The confirmed cases were almost exclusively those in the hospitals and reached 2,264 so far.) While the government restricted parents’ access to these homes, it allowed positive-tested workers and professionals to continue working there. The distribution of cases in Germany (Figure 2) shows a higher incidence in the working-age population than in the 60–80 age group and a steep increase in the age groups at retirement homes. The moderately higher incidence in females could mirror their higher presence in social services. Occupational risks for COVID-19 were recognized early on.^20^ Finally, a large array of publications suggest that there is a disproportionate COVID-19 burden on working classes in every country where it has been investigated – for example, in France^21^ and Korea.^22^
- With 1,534,605 cases and 151,429 deaths at May 10, Europe had the world’s highest burden of COVID-19.

**Table 1.** Country-Specific COVID-19 Case Fatality Rate Among Confirmed Cases (as of June 6, 2020) and Excess Deaths Compared to the Same Periods in Some European Countries.^a^

	CFR	Excess Deaths
France	19.02	34% (March 16–April 26)
Belgium	16.24	51% (March 16–May 10)
Italy	14.4	49% (in March)
United Kingdom	14.21	56% (March 14–May 15)
Spain	11.24	72% (March 16–May 10)
Germany	4.71	3% (March 16–April 26)

Abbreviation: CFR, case fatality rate.

^a^Data obtained from Our World in Data^26^ and Wu et al.^27^

**Table 2.** Significant Clusters Being Investigated in New Zealand as of May 5, 2020.^a^

Non-Work Environment		Work Environment	
Wedding	98	Marist College	94
Private function	39	Hospitality venue	76
Community	30	Residential care facility for the elderly	55
Ruby Princess Cruise ship cluster	23	Residential care facility for the elderly	43
Group travel to United States	16	World Hereford Conference	38
Group travel to United States	16	Residential care facility for the elderly	20
Wedding	13	Residential care facility for the elderly	15
		Residential care facility for the elderly	13
		Workplace	10
Total	235		364

^a^Data from obtained from New Zealand Ministry of Health.^18^

### Rift Valley Fever

- Rift Valley Fever^28^ (RVF) surveillance, tracking, contact tracing, and isolation are effective.
- R0 is low, the human-to-human transmission being quite limited.
- The biggest epidemics were comparatively modest (Sudan, 2007, 738 cases) because epidemics tend to be self-limiting.
- CFR was 4% in Sudan.
- RVF mortality is low.
- RVF-specific mortality primarily concerns workers in contact with dead animals.
- RVF does not occur in Europe. Although currently confined to Africa and the near East, this disease causes concern in countries in temperate climates where both hosts and potential vectors are present, such as the Netherlands.^29^

### Rabies

- Rabies^30,31^post-exposure vaccination and immunoglobulin are effective. Prevention with dog immunization is effective.
- There is practically no human-to-human rabies transmission.
- Worldwide, the disease burden is significant, with 15 million cases per year.
- Without treatment, rabies is always fatal (CFR = 100%).
- Worldwide, mortality was 17,400 per year in 2015.
- There are no occupational clusters.
- In 2013, no cases were reported in the European Union, but 3 were reported in the European Economic Area (Russian Federation).^32^

### SARS

- No vaccine and treatment exist for SARS.^33,34^ However, tracing, tracking, and quarantine are effective in limiting epidemics.
- The R0 is 3.5^5^ (2–4 then R_e_ became 0.4 in 2003).
- During the 2003–2005 epidemic, there were 8,422 cases, all in Asia.
- The CFR is 11% for all age groups, but 55% over age 65.
- During the pandemic, 925 deaths occurred. The CFR was 9.6%.^35^
- Occupational concentration is probable in health and social services.
- No cases occurred in Europe.

### MERS

- There is no treatment and no vaccine.^36^
- The R0 is 2.5–7.^37^
- During the 2012–2013 epidemic, 75 severe cases were recorded. The number of mild cases was unknown.
- The CFR was 65%.
- There were 49 deaths during the 2012–2013 epidemic.^35^
- Occupational concentration in health and social services could be expected.
- There have never been MERS cases in Europe.

### Poliomyelitis Type 1, 2, 3

- The vaccine is effective.^38^
- Poliomyelitis is a highly contagious disease (R0 = 6).^5^
- Type 2 was declared eradicated in 1999. There have been no Type 3 cases since 2012. In 2019, 175 cases of Type 1 were reported. One per 200 cases leads to irreversible paralysis.
- Of those with paralysis, 5%–10% die.
- There are no occupational polio clusters.
- Europe was declared polio-free in 2002.^39^

### Hepatitis B

- The vaccine and Tenofovir treatment are effective.^40^
- Hepatitis B (HBV) is highly contagious. Its R0 is 5 when there is no intervention.
- In 2015, 257 million were infected worldwide, of which 27 million were diagnosed and 4.5 million treated.
- 4,2/°° of acute viral hepatitis cases in Italy died between 1995 and 2000.^41^
- Worldwide, 887,000 die yearly. Between 1993 and 2012, age-standardized, HBV-related liver mortality rates declined from 0.2 to 0.1 per 100 person-years (*P* < .001).^42^
- Incidence is higher in health care workers than in general population because of exposure to blood products.
- In Europe, 1.6% of the population is infected with HBV. In 2017, 30 European Union/European Economic Area member states reported 26,907 cases. The crude incidence rate was 6.7 cases per 100,000 population. R_e_ is below 1 as the number of new cases continues to decline. In Europe, it causes 56,000 deaths per year.^43^

### Hepatitis C

- “Increased access to highly effective direct-acting antivirals (DAAs) for the treatment of infection with the hepatitis C virus (HCV) is revolutionizing the prospect of ending HCV epidemics,” the World Health Organization (WHO) stated in March 2018. However, there is no vaccine.^44,45^
- Transmission is primarily transmitted during injections, in health care settings, and during transfusion of unscreened blood, but some sexual transmission occurs and it can be passed from infected mothers to infants. HCV becomes chronic in 50% of cases.^46^
- 71 million people have been infected with HCV. Chronic hepatitis results in at least 75% of patients.
- Today, CFR is low. From 2003 to 2013, CFR ranged from 0.3% over 5.7 years to 9.2% over 8.2 years of follow-up in community samples. Among treated patients achieving sustained viral response, liver-specific case fatality was low: up to 1.4% over 11.5 years of follow-up.^47^ In the United States, the mortality rate began to decline in 2014.^48^ New treatments can cure more than 95%.
- Each year, 399,000 die. This number is likely to be largely underestimated.
- Health care workers are especially at risk, due to needle sticks involving HCV-positive blood.
- In Europe, 14 million are chronically infected, and 112,500 die per year from HCV-related cancer and cirrhosis.

### Dengue

- A vaccine is available for 9- to 45-year-old people with a previous episode.^49^
- Aedes Egypti, a mosquito, transmits the disease.
- There are 390 million cases worldwide yearly, of which 96 million are symptomatic. Fewer than 1% are considered severe. 70% of cases are in Asia.
- The CFR is below 1%.
- Worldwide, 4,032 cases died in 2015.
- There is no occupational concentration.
- In Europe, the disease is exceptional. In 2018, 14 autochthonous dengue cases were reported from continental Europe (8 cases in France and 6 cases in Spain).^50^

### Japanese Encephalitis

- Immunization is effective.^51^
- Children are the main transmitters.
- Worldwide, there are 68,000 cases per year.
- The CFR is 30% of severe cases.
- Mortality is 13,600–20,400 per year.
- There is no occupational concentration.
- The disease is not present in Europe.

### West Nile Virus

- Physical protection of workers is effective. There is no treatment (although supportive treatment is available) and no vaccine.^52,53^
- Dead birds transmit the disease. There is no human-to-human transmission.
- One per 150 cases is severe.^54,25^
- CFR varies from 4% to 14% (14% in severe cases among the elderly).
- Regarding mortality, 50 deaths were reported in Europe between January 1 and November 30, 2019.
- Workers in contact with dead animals are the main victims of the virus.
- In 2018, 1,548 cases were reported in Europe.

### Yellow Fever

- The vaccine is effective. No antiviral therapy is available, but supportive treatment is relatively effective. Aedes Egypti control is effective but difficult.^55^
- There is no human-to-human transmission. Epidemics are related to mosquitoes.
- Worldwide, there were 84,000–170,000 severe cases in 2013.
- CFR is 15%–50%.^56^
- Mortality is 29,000–60,000 deaths per year.
- Yellow Fever shows no occupational concentration.
- The disease is exceptional in Europe. For 2017, European Union/European Economic Area countries reported 1 travel-related case of Yellow Fever. The case was reported by the Netherlands with exposure in Suriname.^57^

### Influenza H7N7

- Prevention consists of surveillance. There is no vaccine and the only available treatment is supportive.^58^
- The risk of transmission to the community is low.^59^
- Incidence is extremely low: 89 cases were reported in the Netherlands in 2003.
- CFR was 1 per 89 in 2003.
- Mortality is very low.
- Occupational concentration occurs among poultry workers, farmers, and veterinarians.
- Three cases were declared in Europe in 2013.

### AIDS

**Figure 3.**
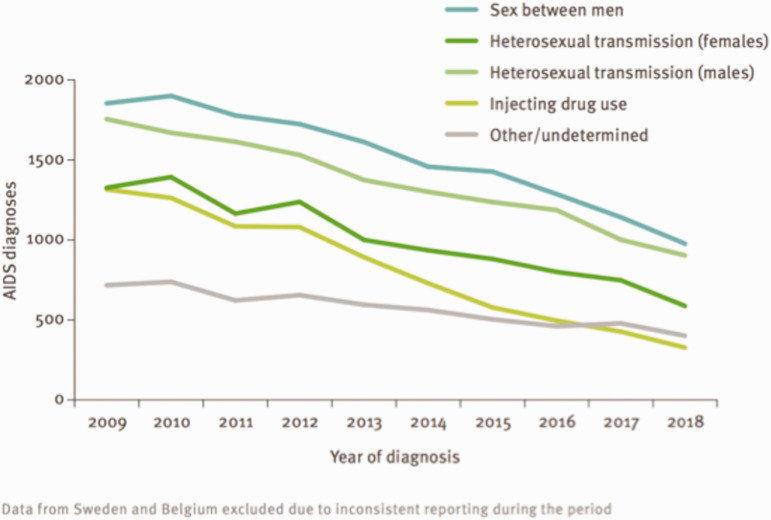
AIDS diagnoses, by transmission mode, European Union/European Economic Area, 2009–2018 (data from Sweden and Belgium excluded). Sources: HIV/AIDS surveillance in Europe 2019 (2018 data), WHO Regional Office for Europe, and European Centre for Disease Prevention and Control.^24^

- Prevention is effective. Diagnosis, treatment, and supportive care are available.^60^
- R0 was 4.6 in Africa in 2014.^61^ In 2008 in Albania, the R0 was 3 for HIV-1A and 1.5 for HIV-1B with, respectively, an average duration of infection of 3 years, while it was 8 and 2.1 with a duration of 10 years.^62^ R0 is 0 when the patient is treated and uses condoms. The 90–90–90 target in Europe aims at 90% of people living with HIV knowing their HIV status, 90% of diagnosed people living with HIV receiving treatment, and 90% of people on treatment achieving viral suppression. R0 in Europe is below 1 since incidence is going down year after year (Figure 3).
- In 2018, there were 1.7 million newly infected, two-thirds of which were in Africa. New infections fell by 45% between 2000 and 2018. In Europe, there were 141,552 new infections in 2018.^63^
- CFR varies according to access to care and care continuity.
- Worldwide, by the end of 2018, 37.9 million had died since the 1980s. In 2018, 770,000 died. Deaths fell by 45% between 2000 and 2018. “3,235 diagnoses of AIDS were reported by 30 [European Union/European Economic Area] countries in 2018 – a crude rate of 0.6 cases per 100,000 population.” In 2018, 822 deaths due to AIDS-related causes were declared in 28 European Union/European Economic Area countries (all but Denmark, Italy, and Sweden).
- Sex workers are the primary occupational victims. In low- and middle-income countries, prevalence was lower among health care workers than in the general population. Only 58 cases of confirmed occupational HIV transmission to health care personnel had been reported in the United States until 2019. An additional 150 possible transmissions have been reported to the Centers for Disease Control and Prevention.^63^
- “In 2018, 141,552 newly diagnosed HIV infections were reported in 50 of the 53 countries in the WHO European Region, including 26,164 from the [European Union/European Economic Area]. This corresponds to a crude rate of 16.2 newly diagnosed infections per 100,000 population. HIV prevalence was highest in the East of the Region (44.8 per 100,000 population), lower in the West and in the [European Union/European Economic Area] (6.0 and 5.6 per 100,000, respectively) and lowest in the Centre (3.3 per 100,000). The number of people newly diagnosed with HIV in the WHO European Region has increased by 22% over the last decade, while the number of new diagnoses among countries in the [European Union/European Economic Area] has declined by 17% since 2009. In 2018, just over half (53%) of those diagnosed with HIV in the European Region were diagnosed at a late stage of infection.”^63^

### Influenza H2N2 (Singapore Influenza)

- A vaccine was discovered in 1957.^64^
- R0 was 1.68 (27) and would be below 1.2 in the case of an epidemic today.^65^
- The incidence of 1957–1959 epidemics and related mortality are speculative.
- The 1957 H2N2 influenza pandemic infection-fatality-ratio would be a quarter of COVID-19 (roughly 9.4 per 1,000), albeit with broad uncertainty.^66^
- The estimated number of deaths was 1.1 million worldwide and 116,000 in the United States.^67^ On average, during 1957–1959, the pandemic-associated excess respiratory mortality rate was 1.9 per 10,000 population (95% confidence interval, 1.2–2.6 cases/10,000 population).^68^
- Health care and social workers are probably the high-risk categories.
- The pandemic mortality rate was the lowest in Europe.

### Influenza H5N1 (Avian Flu)

- If there is no available vaccine, the oseltamivir treatment is effective, as is surveillance.^69,70^ Mortality remains high because delayed initiation of treatment appears to be a key factor, as exemplified by the most recent analysis of data from Indonesia, the experience of Egyptian patients, and pooling of results for the years 2004–2006 from Vietnam, Thailand, Indonesia, and Turkey.^71^
- R0 is very low.
- As far as incidence is concerned, only 861 cases were recorded worldwide between 2003 and 2020.
- The CFR is 60%.
- Between 2003 and 2020, mortality reached only 455.
- Workers in contact with birds (e.g., farmers, workers in poultries, veterinarians) are the most susceptible to being contaminated.
- In Europe, no cases were declared between 2003 and 2020.

### Chikungunya

- There is neither prevention nor treatment available.^72^
- The disease is transmitted by an Aedes-type mosquito. There is no human-to-human transmission.
- Overall, 1.9 million cases have been declared in Asia since 2005.
- The CFR is 0, but the disease is a death cofactor in the elderly.
- Mortality is almost zero.
- The disease has no occupational concentration.
- The disease is not present in Europe.^73^

### Ebola

- As far as prevention is concerned, the rVSV-ZEBOV vaccine was successfully used in the Democratic Republic of the Congo 2018–2019 outbreak. Surveillance, contact tracing, and quarantine are effective. A multidrug therapy is being tested. Supportive treatment is moderately effective.^74^
- R0 has been 1 to 1.3^75^ and even 1.5.^76,77^
- With regard to incidence, 29,000 cases occurred in Sierra Leone, Liberia, and Guinea during the 2014–2016 epidemic. Of these, 27% of infections were asymptomatic.^78^
- CFR is 50% (25%–90%).
- There were 11,500 deaths during the Sierra Leone, Liberia, and Guinea epidemic in 2014–2016.
- The greatest risk of secondary attack is in nursing care and health care services in general.
- Only a few imported cases occurred in Europe (primarily health professionals contaminated in Africa and treated in Europe).

### Marburg Hemorrhagic Fever

- There is no prevention and no treatment.^79^
- The R0 is 1.59.^80^ Primarily bats transmit the disease, but there is some human-to-human transmission.
- In 1967, 29 cases occurred in Germany, initially as a result of laboratory contamination. During the Angola epidemic, 374 cases were declared in 2005. Epidemics in humans are rare because of the relatively small reproduction number and long generation time.
- CFR is 80%.
- Mortality rate is uncertain: 1 death in Germany in 1967, 329 deaths in Angola in 2005.
- Occupational concentration occurs primarily among health care workers.
- In Europe, there has been no case in more than half a century.

### Smallpox

- The vaccine is effective.^81^
- The disease had a primarily inter-human transmission, but it could also be spread through direct contact with infected bodily fluids or contaminated objects, such as bedding or clothing. R0 was 4.5.
- With regard to incidence, the last case succeeded in Somalia in 1977. The disease was declared eradicated in 1980.
- CFR was 4.5.
- There was no occupational concentration.

### Lassa Fever

- There is no vaccine. Ribavirin is an effective treatment if taken early.^82^
- Infection is acquired through contact with an infected rodent, but person-to-person transmission is possible (R0 was 1.06–1.62 in Nigeria, 2018).^83^ Lab infections exist.
- The virus is endemic in West Africa, with 100,000–300,000 cases per year,^84^ of which 15% are severe (of these, 25% of cases are deaf if they survive).
- CFR is 1%.
- With respect to mortality, 5,000 deaths are recorded each year worldwide.^83^
- Occupational concentration occurs in health care workers.
- The disease is not present in Europe.

## Discussion

The list of studied viral pathologies may be incomplete from a public health significance perspective. The provided statistics only give an order of magnitude, and this is how we used them to draw our conclusions, especially since it is too early to estimate the R0 and CFR of COVID-19. In addition, countries declare the disease heterogeneously. Some demand a test, others suggestive symptoms, while others do not count deaths in nursing homes. Data on the period-to-period difference in mortality between 2020 and previous years are only beginning to emerge; to enable inter-country comparisons, the COVID-19 mortality will have to be assessed through excess mortality rates, by comparing overall country-specific, monthly mortality rates to the previous 5-year average mortality rate of the same month.

Pooled mortality estimates from the EuroMOMO network continue to show a markedly increased level of excess all-cause mortality overall for participating European countries, coinciding with the current COVID-19 pandemic. This overall excess mortality is driven by a very substantial excess mortality in some countries, while other countries have had no excess mortality. The mortality excess is primarily seen in the age group of ≥65 years, but also in the age group of 15–64 years. For the EuroMOMO network as a whole, from Week 10 in 2020 and as of Week 18, there were 149,447 excess deaths estimated in total, including 137,524 in the age group ≥65 years and 11,573 in the 15–64 years age group. This time period includes part of the influenza season as well as the start of the COVID-19 pandemic.^85^

Comparisons between mortality of epidemic diseases (such as COVID-19) and chronic ones (such as HCB or HCV) can be misleading, especially because we do not know yet if COVID-19 will provoke new waves or possibly mutate and be periodically recurrent.

In general, data quality is much lower in low- to middle-income countries, as are CFRs, because of limited access to high-quality health care. Some diseases, such as HVB and HVC, are known to be heavily underdiagnosed. With regard to COVID-19, several countries are reluctant, for political reasons, to publish honest data that allows for comparison.

As we are still in the middle of the first pandemic of COVID-19, the comparison should be made with caution.

## Conclusion

Table 3 summarizes our findings.

**Table 3.** Summary of Virus Characteristics Comparison to COVID-19 Features.^a,b^

	Prev. Treat.	R0	Incidence	CFR	Mortality	Occupational Concentration	Cases in Europe
COVID-19	−	2.5–>0.3	3,623,803 cases worldwide;1,429,897 cases in Europe	4.24–13.76	256,880 deaths worldwide; 143,898 deaths in Europe	Social services; other work clusters	+
Rift Valley Fever	−	0	Max 738/epidemic	4%		Farmers, veterinarians	−
Rabies	+	0	15M/year	100%	17,400/year (2015)	–	− in EU
SARS	−	3.5–>0.4	8,422 in 2002–2004 epidemic	11% overall; 55% > 65 years old	925 in 2002–2004 epidemic	Unknown	−
MERS	−	<1	75 severe cases in 2012–2013	65%	49 in 2012–2013; MR = 34%?	–	−
Polio 1, 2, 3	+	6	175 cases P1 in 2019 (1/200)	5_10% of paral.	0	–	−
Hepatitis B	+	5	275M 2015	0.1/100 PY	887,000/year; 56,000 deaths/year in Europe	HCW	+
Hepatitis C	+	?	71M worldwide; 14M in Europe	0.3–9.2% (2003–2013); today, under treatment, <1.5%	399,000/year worldwide; 112,500 in Europe	HCW	+
Dengue	+/−	0	96M/year symptom.	<1%	4,032 (2015)	–	exceptional
Japanese Encephalitis	+		68,000/y	30% of severe cases	13,600–20,400/year	–	−
West Nile Fever	−	0	?	4%–14%	50 deaths in Europe 2019	Contact with dead animals	1,508 (2018)
Yellow Fever	+	0	84,000–170,000 severe cases 2013	15%–50%	29,000–60,000/year	–	Exceptional
Influenza H7N7	−	<1	89 cases in Holland 2003	1.12% Holland 2003	1 death in Holland	Farmers, poultry workers	Exceptional
AIDS	+	<1 in Europe; 4.6 in Africa	1.7M 2018; 2/3 Africa; 141,552 European Union	Variable	770,000 deaths 2018; 2,176 deaths 2014	Sex workers	+
Influenza H2N2	+	1.2–1.68	1,957 epidemic. N = ?	?	1.1M deaths worldwide; 19/100,000	–	+
Influenza H5N1	+	0		60%	455 deaths 2003–2020	Contact with birds	−
Chikungunya	−	0	1.9M in Asia since 2005	0	0	–	−
Ebola^c^	+	1–1.5	29,000 in 2014–2016 epidemic	50%	11,500 in 2014–2016 epidemic	HCW	−
Marburg Hem. Fever^c^	−	1.59	374 cases Angola 2005	80%	329 deaths Angola 2005	–	−
Smallpox^c^	+	4.5	–	65% in unvaccinated	–	–	−
Lassa Fever^c^	+	1.06–1.62	100,000–300,000/year	1% and	5,000/year	HCW	−

Abbreviation: CFR, case fatality rate; HCW, health care workers.

Incidence data: Plain numbers express the worldwide number of cases in 1 year unless indicated otherwise. Treatment prevention is noted (–) when only surveillance, detection, contact tracing, and isolation are effective, but no vaccine or treatment is.

^a^All diseases in Group 3 except as indicated.

^b^M = millions.

^c^This disease is in Group 4.

Among the studied diseases in Group 3, HVB and HVC (Hepatitis B and C), H2N2, and HIV (AIDS) represent a public health risk to E.U. citizens. The others are either absent or exceptional in Europe. HBV, HBC, H2N2, and HIV, however, have an effective treatment and/or prophylaxis – unlike COVID-19. On this criterion, the key one that distinguishes Groups 3 and 4, COVID-19 should be classified in Group 4.

Another key reason is that it represents a bigger public health threat to E.U. workers, professionals, and population: In Europe, COVID-19 has killed in 3 months more than 65 times the number of people killed in 1 year by AIDS, 3 times more than HBV, and 2.3 times more than HBC. H2N2 has been silent for 60 years. All Group 3 viruses, present in Europe or not, have a total mortality inferior to COVID-19. Poliomyelitis, HBV, and AIDS have a higher R0 in the rest of the world but not in Europe. Untreated, 5 type 3 viruses have a CFR higher than COVID-19.

By contrast with COVID-19, 3 of the 4 diseases belonging to Group 4 have an effective treatment and/or vaccine. On this criterion alone, COVID should be classified within Group 4. Furthermore, the COVID-19 R0 exceeds the Ebola R0 in early epidemics – that is, in situations unaffected by prophylactic interventions.

What would be the consequences on COVID classification if Ebola, Lassa, and Marburg fevers were actually found in Europe? Basically, our conclusion would remain valid because the COVID-19 R0 is superior to the R0 of these pathologies and their incidence is so low that although their CFR is superior, the mortality of these 3 diseases would remain vastly inferior to the COVID-19 mortality.

While most of the studied viruses concentrate on health care workers and workers in contact with animals, COVID-19 has a wide distribution that concerns jobs in essential sectors (nurses, midwives, physicians, caregivers, child minders, home helpers, paramedical professions, social work and guidance professionals, army, police, firefighters, cashiers, etc.). Many more, particularly precarious workers, are at risk in non-essential, recreational sectors, such as restaurants, gyms, and shopping malls.

In conclusion, COVID-19 characteristics justify its classification in Group 4. Furthermore, and importantly, with effective protection of workers and professionals (particularly in health care services, nursing homes, and social services, although not limited to these sectors), the European Union would not only improve health in work environments, but also activate a mechanism key to reducing COVID-19 transmission, morbidity, and mortality in the general population. In public health terms, the strongest worker protections would be a highly cost-effective intervention. In economic terms, it would probably be a highly cost-beneficial intervention.

Admittedly, the availability of a new vaccine or treatment would imply a change in the proposed classification.
